# Parvovirus B19 Infection in Pregnancy—Course of the Disease, Fetal Complications and Management Tools: A Case Series and Literature Review

**DOI:** 10.3390/children11091037

**Published:** 2024-08-24

**Authors:** Olga Olejniczak, Jakub Kornacki, Daniel Boroń, Paweł Gutaj, Rafał Iciek, Ewa Wender-Ożegowska

**Affiliations:** 1 Department of Reproduction, Chair of Reproduction and Perinatal Medicine, Poznan University of Medical Sciences, 61-701 Poznan, Poland; 2 Doctoral School, Poznan University of Medical Sciences, 60-701 Poznan, Poland

**Keywords:** parvovirus B19, fetal anemia, cordocentesis, intrauterine transfusion, fetal hydrops, mirror syndrome

## Abstract

Parvovirus B19 is a virus that causes a common and usually harmless infection in both children and adults. If the virus is transmitted transplacentally during pregnancy, it can have serious consequences for both the pregnant woman and the fetus. Potential complications include severe fetal anemia, which can lead to intrauterine fetal death. A common ultrasound finding in fetuses affected by parvovirus B19 is fetal edema, which is associated with a poor prognosis. Additionally, a rare but serious complication in pregnant women with parvovirus B19 infection is mirror syndrome. The diagnosis of parvovirus B19 infection during pregnancy necessitates close monitoring of the fetal condition. If fetal anemia is suspected, intrauterine transfusion is indicated to increase fetal survival. This study presents eight cases of parvovirus B19 infection in pregnant women, highlighting the various maternal-fetal complications encountered, along with diagnostic and treatment strategies.

## 1. Introduction

Parvovirus B19 is a single-stranded DNA virus responsible for erythema infectiosum, a common disease in children characterized by its pathognomonic symptom of facial erythema. The virus is typically transmitted through respiratory secretions and hand-to-mouth contact, but it can also spread via blood transfusions and vertical transmission from mother to fetus [1].

The course of parvovirus B19 infection can vary significantly. In adults, the most common symptoms are joint pain and fever. However, in rare cases, serious complications such as severe anemia, aplastic crisis, and myocarditis may occur. Additionally, many individuals infected with parvovirus B19 are asymptomatic, making it challenging to avoid potential sources of infection [2].

According to current scientific data, parvovirus B19 infection confers lifelong immunity in immunocompetent individuals [3]. Women without a previous history of erythema infectiosum are at high risk of transmitting parvovirus B19 to the fetus if they become infected during pregnancy. Transplacental transmission of parvovirus B19 occurs in approximately 17–33% of cases, regardless of whether the maternal infection is symptomatic [4,5].

Pregnancy does not alter the course of parvovirus B19 infection, but the infection can significantly impact pregnancy, particularly fetal development [6]. In most cases, fetuses affected by parvovirus B19 are asymptomatic and recover spontaneously [7]. However, serious complications such as miscarriage, fetal hydrops, and intrauterine fetal death can also occur [8].

Parvovirus B19 is one of the most common causes of non-immune hydrops fetalis. The pathogenesis of hydrops is likely related to the transplacental transfer of the virus, which has an affinity for erythrocyte progenitor cells. Parvovirus B19 infection induces apoptosis in these infected cells. Combined with the short half-life of fetal erythrocytes, this leads to reticulocytopenic fetal anemia [9]. Another possible mechanism for hydrops is viral myocarditis, which impairs myocardial contractility and results in heart failure [10].

The incidence of fetal hydrops in pregnancies complicated by parvovirus B19 infection is estimated at 2.9%. The risk of hydrops fetalis is higher in the early stages of pregnancy and decreases as pregnancy progresses [11]. The occurrence of fetal hydrops is strongly associated with adverse perinatal outcomes [8].

Detecting ultrasonographic changes characteristic of parvovirus infection, monitoring Doppler flows in the middle cerebral artery, and taking a detailed history of possible parvovirus infection in a pregnant woman are fundamental for proper fetal supervision [12].

In cases of fetal anemia caused by parvovirus B19 infection, intrauterine blood transfusion is recommended. Alternatively, if the fetus is at term or near term, delivery may also be considered as a form of treatment [13].

In this case review, we present eight cases of parvovirus infection in pregnant women at various stages of pregnancy, each with distinct obstetric outcomes.

## 2. Materials and Methods

This case study involved a total of eight pregnant women hospitalized at the Gynecology and Obstetrics University Hospital in Poznan, Poland, the tertiary centre for fetomaternal medicine, between February 2024 and the end of April 2024 due to Parvovirus B19 infection and associated pregnancy complications.

Maternal characteristics, fetal ultrasound examinations, fetal blood sampling results, details of intrauterine transfusion (IUT) procedures, and perinatal outcome data were analyzed.

In all cases, fetal hydrops was detected during ultrasound examinations. Cordocentesis with intrauterine transfusion was performed in four cases. Unfortunately, intrauterine fetal death occurred in four out of the eight fetuses.

Each cordocentesis procedure was performed by a specialist in maternal-fetal medicine. The primary objective in every case was to puncture the umbilical vein (either at the free loop or cord insertion) to obtain a blood sample for a complete blood count and administer rocuronium based on the estimated fetal body weight. Subsequently, blood transfusion was initiated without removing the needle from the vessel. In instances where umbilical vein puncture was technically unfeasible, blood was administered intraperitoneally. The volume of blood transfused was adjusted according to the severity of anemia, calculated using the donor hematocrit (75%), initial fetal hematocrit, final fetal hematocrit (~45%), and estimated fetal weight.

A table comparing the key characteristics of the cases presented in this study is provided below (Table 1).

## 3. Cases

### 3.1. Case 1

A 36-year-old woman, gravida 2, para 0,1,0, was admitted to the hospital at 15 weeks of gestation for further diagnostics of fetal hydrops detected during first-trimester screening. The patient had no chronic diseases and did not experience any symptoms of infection in the weeks leading up to the prenatal examination. She was unable to recall any contact with a person exhibiting typical symptoms of parvovirus B19 infection. However, a potential risk factor for parvovirus infection was identified: her preschool-age child attended kindergarten, where cases of erythema infectiosum had been reported, although the child did not display any symptoms of infection.

Upon admission to the hospital, ultrasound assessment identified a singleton gestation with the fetus showing characteristic signs of hydrops, such as ascites, hydrothorax, and subcutaneous tissue edema. The placenta was observed to be thickened and situated anteriorly within the uterus. Doppler examination revealed indications of fetal anemia, with a peak systolic velocity (PSV) in the middle cerebral artery (MCA) measuring 62.00 cm/s (2.99 MoM).

During hospitalization, amniocentesis was conducted, collecting 30 mL of amniotic fluid for Parvovirus B19 DNA PCR testing and microarray analysis. The procedure was performed without complications. Blood samples were also taken from the patient for serological testing for Parvovirus B19. The patient was discharged with an ongoing pregnancy and readmitted after 5 days for result discussion and intrauterine transfusion.

Laboratory tests revealed the presence of both IgG and IgM antibodies against parvovirus B19 in the maternal blood. Additionally, PCR testing of the amniotic fluid confirmed fetal parvovirus B19 infection. Microarray analysis did not detect any chromosomal abnormalities.

Upon readmission, an ultrasound examination revealed no fetal heartbeat and worsening fetal hydrops. Despite recommendations for termination of the pregnancy, the patient opted against it and was discharged with instructions to follow up with her attending obstetrician.

### 3.2. Case 2

A 30-year-old woman, gravida 2, para 1,0,0, was referred to the hospital due to suspected fetal heart defect and signs of fetal hydrops identified during a routine ultrasound examination at 23 weeks of gestation.

An ultrasound examination conducted at the hospital revealed generalized fetal hydrops. No other fetal anomalies were observed, and the amniotic fluid volume was within normal limits. The peak systolic velocity of the middle cerebral artery was measured at 65.31 cm/s, corresponding to 2.17 MoM.

The patient experienced cold-like symptoms 4 weeks prior to admission to the clinic. Parvovirus B19 infection was confirmed by determining the presence of specific IgG and IgM antibodies in the patient’s serum.

The cordocentesis with intrauterine transfusion was scheduled for the following day. On the day of the planned procedure, the patient reported no fetal movements. Subsequent ultrasound examination revealed no fetal heart activity.

The patient consented to labor induction. A male stillborn baby weighing 1150 g was delivered. The birth was uncomplicated. The patient was discharged in good general condition.

### 3.3. Case 3

A 35-year-old patient in her 25th week of her third pregnancy, following two uncomplicated vaginal deliveries, was admitted to the hospital due to abnormalities found during a routine ultrasound examination.

No signs of infection were observed in the month before admission. One of the patient’s children attended a kindergarten where cases of erythema infectiosum had been reported.

Upon admission to the hospital, ultrasound examination revealed extensive fetal hydrops characterized by severe ascites, hydrothorax, and significant fluid accumulation in the pericardium. The peak systolic velocity (PSV) in the middle cerebral artery (MCA) measured 63.00 cm/s, corresponding to 1.94 MoM.

Additionally, the patient presented with oligohydramnios. A rapid test for detecting amniotic proteins in a vaginal swab yielded inconclusive results. Prophylactic antibiotic therapy consisting of azithromycin, ampicillin, and amoxicillin was initiated to prevent infection in case of premature rupture of membranes. Steroid therapy was also administered as a precaution against premature birth.

In the early morning of the scheduled day for cordocentesis with intrauterine transfusion, the patient reported an absence of fetal movement. Subsequent ultrasound examination confirmed intrauterine fetal death.

Labor was induced. A male stillborn baby weighing 1380 g was delivered. The birth was complicated by peripartum hemorrhage, necessitating a transfusion of two units of packed red blood cells post-delivery. The patient was discharged in good general condition on the fourth day of the postpartum period.

### 3.4. Case 4

A 28-year-old patient in the 20th week of her first pregnancy was admitted to the hospital due to fetal hydrops detected during a routine ultrasound check-up. The patient did not report any recent symptoms of infection, nor had she been in contact with anyone suspected of parvovirus B19 infection.

Ultrasound performed on the first day of hospitalization confirmed the diagnosis of fetal hydrops, revealing pleural effusion, ascites, subcutaneous tissue edema, and cardiomegaly. Doppler ultrasound indicated fetal anemia, with a peak systolic velocity of the middle cerebral artery measuring 60.43 cm/s (2.37 MoM).

Cordocentesis with intrauterine transfusion was performed. Unfortunately, the fetal blood count could not be determined due to technical issues (clot formation in the collected sample). Nevertheless, it was decided to proceed with intrauterine blood transfusion. A total of 30 mL of fluid was extracted from the fetal abdominal cavity, and 20 mL of type O-negative irradiated blood was administered intraperitoneally. Subsequently, rocuronium was administered based on the estimated fetal body weight, followed by the administration of 18 mL of blood into the umbilical vein (free loop). At the conclusion of the procedure, 30 mL of amniotic fluid was collected for parvovirus B19 PCR and microarray testing.

The following day, a follow-up ultrasound showed a PSV MCA of 47.82 cm/s (1.82 MoM). In subsequent ultrasound examinations, the PSV MCA gradually increased. When the peak systolic velocity of the middle cerebral artery reached 54.3 cm/s (2.02 MoM), it was decided to perform another intravenous transfusion.

The second intrauterine blood transfusion was conducted during the 21st week of pregnancy. A fetal blood sample was obtained for a complete blood count, and rocuronium was administered. The test revealed an initial hematocrit of 16%, followed by 17 mL of blood transfusing into the umbilical vein.

The day after the procedure, the peak systolic velocity (PSV) of the middle cerebral artery (MCA) was measured at 41.00 cm/s (1.51 MoM).

One week after the second transfusion, the MCA PSV increased again to 65.26 cm/s (2.23 MoM). In the 23rd week of pregnancy, the third cordocentesis with intrauterine transfusion (IUT) was performed. The fetal blood sample taken at the beginning of the procedure showed a hematocrit of 14%, with fetal leukocytes undetectable. Following puncture of the free loop of the umbilical vein and administration of rocuronium, 25 mL of blood was transfused. The day after the IUT, the PSV of the MCA was 64.01 cm/s (2.14 MoM), and 2 days after the procedure, it measured 49.87 cm/s (1.66 MoM).

**Figure 1:**
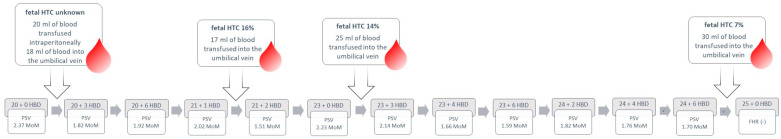
Case 4: Doppler results and treatment strategy presented in a flowchart.

Doppler results and the timing of the intrauterine transfusions are presented in Figure 1.

**Figure 2:**
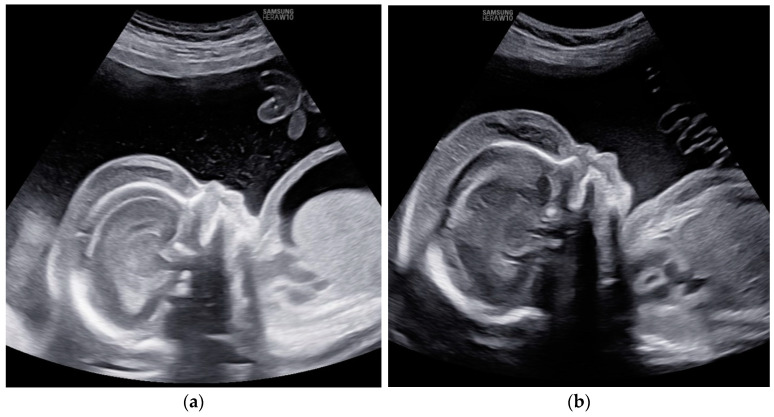
Case 4: Edema of fetal subcutaneous tissue; no signs of resolution of hydrops; (**a**) before first intrauterine transfusion, 20 weeks of gestational age; (**b**) after third intrauterine transfusion, 24 weeks of gestational age.

Despite a slight improvement in Doppler findings, no resolution of fetal hydrops was observed (Figure 2).

Due to the patient’s decision to continue therapy despite the poor prognosis for fetal survival, a course of steroid therapy was administered to accelerate fetal lung maturation.

Due to the absence of improvement in the ultrasound image, it was decided to perform the fourth intrauterine transfusion (IUT) at 24 weeks of pregnancy. The fetal blood count revealed a hematocrit of 7% and a white blood cell count of 3.31 [G/L]. After puncturing the umbilical vein, rocuronium was administered, and 30 mL of blood was transfused.

The day after the fourth intrauterine transfusion (IUT), an ultrasound examination revealed the absence of fetal heart activity. Labor was induced, resulting in the delivery of a male stillborn baby weighing 1385 g. The birth was complicated by peripartum hemorrhage, and following delivery, the patient required a transfusion of two units of packed red blood cells.

### 3.5. Case 5

**Figure 3:**
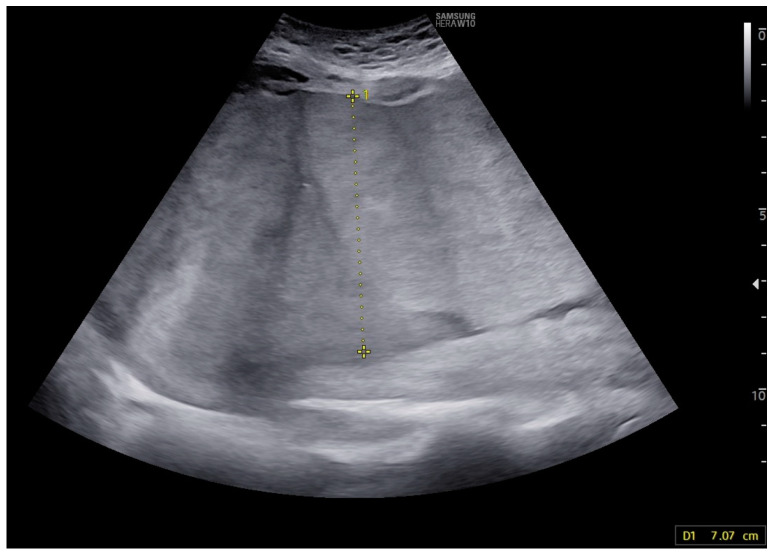
Case 5: Thickening of the placenta; after second intrauterine transfusion, 25 weeks of gestation.

A 32-year-old woman, gravida 2, para 1,0,0, was referred to the hospital due to abnormalities found during a second-trimester ultrasound examination. The findings included placental thickening (Figure 3), subcutaneous tissue edema, ascites, pericardial effusion, and cardiomegaly. The peak systolic velocity of the middle cerebral artery was measured at 78.00 cm/s (2.6 MoM).

The patient reported that 2 weeks before admission to the hospital, her 4-year-old son exhibited symptoms of erythema infectiosum. She underwent a serological test, which confirmed the presence of IgG and IgM antibodies against parvovirus in her serum.

It was decided to perform a cordocentesis with intrauterine transfusion the next day. Fetal blood count revealed a hematocrit of 11%. A total of 27 mL of type O-negative blood were transfused into the umbilical vein (free loop) following the administration of an appropriate dose of rocuronium. The next day, an ultrasound examination showed a decrease in the peak systolic velocity (PSV) of the middle cerebral artery to 57.60 cm/s (1.9 MoM). On the ninth day of hospitalization, a slight decrease in the amount of amniotic fluid was noted (maximum vertical pocket [MVP] 3.2 cm). The patient did not report rupture of membranes.

Nine days after the first intrauterine transfusion, it was decided to repeat the treatment and perform the procedure again. The fetal blood count showed a hematocrit of 17%. A total of 23 mL of blood was transfused into the peritoneal cavity.

An ultrasound performed the day after the second intrauterine transfusion (IUT), at 24 weeks of gestation, revealed oligohydramnios (amniotic fluid index [AFI] 9 cm) and slight improvement in Doppler flow in the middle cerebral artery (PSV MCA 57 cm/s, 1.79 MoM), but fetal hydrops did not resolve. Due to a significant decrease in amniotic fluid, a rapid test for detecting premature rupture of fetal membranes was conducted, yielding a positive result. Vaginal swabs were collected for culture. Antibiotic therapy was initiated based on recommendations for treating premature rupture of membranes and was modified according to the culture results and the antibiogram.

In the following days of hospitalization, elevated blood pressure readings were observed in the patient. Blood tests revealed a significantly elevated sFlt-1/PlGF ratio (264). Antihypertensive treatment was initiated, consisting of methyldopa at a daily dosage of 2000 mg, amlodipine at a daily dosage of 20 mg, and metoprolol at a daily dosage of 100 mg.

The patient began to show signs of fluid retention and oliguria. Protein loss was assessed through a 24-h urine collection, revealing significant proteinuria of 6.59 g per 24 h.

The patient’s clinical presentation raised suspicion of mirror syndrome, characterized by maternal symptoms mirroring those observed in the fetus.

**Figure 4:**
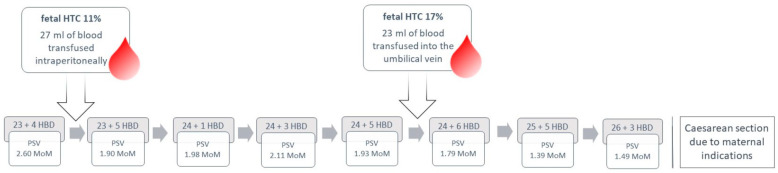
Case 5: Doppler results and treatment strategy presented in a flowchart.

The fetal condition was assessed through ultrasound examinations, which revealed no signs of decrease in fetal edema. Doppler results showed stability, with the peak systolic velocity (PSV) of the middle cerebral artery (MCA) fluctuating between 1.5–2.0 MoM. The end-diastolic flow in the umbilical artery was positive, and the pulsatility index of the umbilical artery was within the normal range. Doppler findings and the timing of intrauterine transfusions are illustrated in Figure 4.

In the 25th week of pregnancy, steroids were administered due to the high risk of preterm delivery.

On the 22nd day of hospitalization, the patient reported upper abdominal pain, and an ultrasound examination revealed the presence of fluid in the mother’s abdominal cavity. Due to the deterioration of the patient’s clinical condition, a planned cesarean section was performed to terminate the pregnancy at 26 weeks of gestation.

A male newborn was delivered in critical condition, weighing 910 g, with Apgar scores of 2, 4, 4, and 4. The newborn died on the fifth day of life.

The patient was monitored in the hospital for 7 days after delivery. During this period, her clinical condition steadily improved: normal diuresis resumed, peripheral edema resolved, and the dosage of antihypertensive medications was reduced.

The patient was discharged from the hospital in good general condition. On the day of discharge, she required continuation of antihypertensive treatment.

### 3.6. Case 6

A 28-year-old pregnant woman in her 24th week of an otherwise uncomplicated pregnancy was admitted to the hospital due to fetal hydrops (including ascites, pleural effusion, pericardial effusion, and subcutaneous tissue edema) detected during a routine ultrasound examination in the 23rd week of gestation.

After admission to the hospital, ultrasound confirmed the presence of the aforementioned conditions, and the measurement of the peak systolic velocity (PSV) of the middle cerebral artery (MCA) was 89 cm/s (3.02 MoM).

The first intrauterine transfusion was performed immediately upon admission, during the 24th week of pregnancy. The fetal blood analysis revealed a hematocrit level of 6%. Initially, 20 mL of type-O negative, irradiated blood was administered intraperitoneally. However, during the procedure, the needle dislodged from the vessel lumen, causing the umbilical cord to collapse, which prevented further puncturing attempts. Consequently, an additional 12 mL of blood was transfused into the peritoneal cavity. Following the transfusion, the fetal heart rate measured 198 beats per minute.

The following day, a slight improvement in the middle cerebral artery was observed, with the PSV MCA decreasing to 81.30 cm/s (2.60 MoM).

A few days after the initial successful transfusion, laboratory tests conducted upon admission to the hospital confirmed the presence of acute parvovirus B19 infection.

The second cordocentesis with intrauterine transfusion (IUT) was performed at 25 weeks of gestation. The fetal blood count showed a hematocrit of 13%. The procedure was uneventful, and 32 mL of blood was transfused into the umbilical vein. The day after the second IUT, Doppler results showed significant improvement, with the PSV MCA measuring 39.9 cm/s (1.23 MoM).

In the late hours following the second intrauterine transfusion, the patient experienced symptoms including chills, fatigue, and a fever reaching 38 °C. A blood sample was promptly collected for culture, and broad-spectrum antibiotic therapy was initiated. Inflammatory markers were notably elevated: CRP was 21.63 mg/dL, PCT measured 0.63 ng/mL, and IL-6 was 27.07 pg/mL.

The next day, the fever spiked to 39 degrees Celsius, with CRP rising to 75.46 mg/dL and IL-6 at 21.36 pg/L. Antibiotic treatment was continued, and the patient received antipyretic medications (paracetamol). By the third day of antibiotic administration, the CRP levels decreased to 51.76 mg/dL, followed by a further reduction to 21.92 mg/dL the subsequent day. Blood culture results returned negative. The patient’s overall condition improved steadily, with her body temperature returning to normal. Ultrasound assessments indicated that the peak systolic velocity (PSV) of the middle cerebral artery (MCA) remained below 1.5 MoM, and fetal hydrops showed gradual improvement.

The patient was discharged after completing the antibiotic therapy.

**Figure 5:**
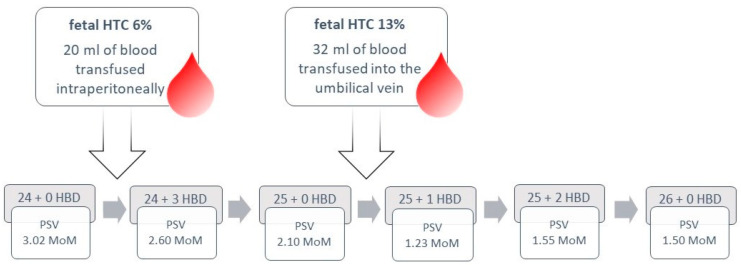
Case 6: Doppler results and treatment strategy presented in a flowchart.

Subsequent ultrasound examinations were conducted in the ensuing weeks. At 27 weeks of pregnancy, the peak systolic velocity (PSV) of the middle cerebral artery (MCA) measured 58.31 cm/s (1.66 MoM), which decreased to 55.00 cm/s (1.49 MoM) by 28 weeks of gestation. Doppler results and the timing of the intrauterine transfusions are illustrated in Figure 5.

**Figure 6:**
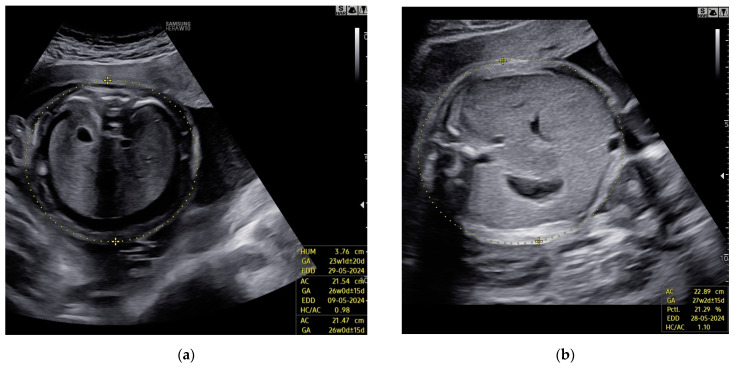
Case 6: Total resolution of ascites (**a**) before first intrauterine transfusion, 24 weeks of gestational age; (**b**) 2 weeks after second intrauterine transfusion, 28 weeks of gestation.

Also, a total resolution of fetal hydrops was observed (Figure 6).

### 3.7. Case 7

**Figure 7:**
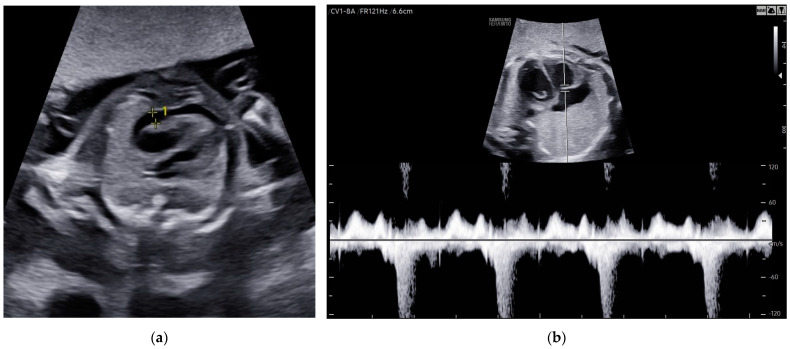
Case 7: Abnormalities in the fetal heart ultrasound: (**a**) pericardial effusion before the first intrauterine transfusion, 21 weeks of gestation; (**b**) regurgitation of the mitral valve and total resolution of pericardial effusion, 2 weeks after the second intrauterine transfusion.

A 29-year-old woman, in the 21st week of her second pregnancy, was referred to the hospital due to anomalies detected during the anatomy scan—specifically, fetal hydrops accompanied by ascites and pericardial effusion (Figure 7a).

At admission, the peak systolic velocity (PSV) of the middle cerebral artery (MCA) was 52.00 cm/s (1.91 MoM). On the second day of hospitalization, cordocentesis with intrauterine transfusion (IUT) was performed. Fetal blood analysis revealed a hematocrit of 15%, and subsequently, 15 mL of blood was transfused into the umbilical vein.

The PSV of the MCA measured the day after the transfusion showed a significant decrease to 27.48 cm/s (1.00 MoM).

Despite a slight regression of fetal edema and stable flow in the middle cerebral artery (MCA) (with a PSV of 37.00 cm/s, corresponding to 1.26 MoM, 2 weeks after the first IUT), another intrauterine transfusion was performed at 23 weeks of pregnancy. The fetal hematocrit determined during cordocentesis was 32%, following which 10 mL of blood was transfused into the umbilical vein.

**Figure 8:**
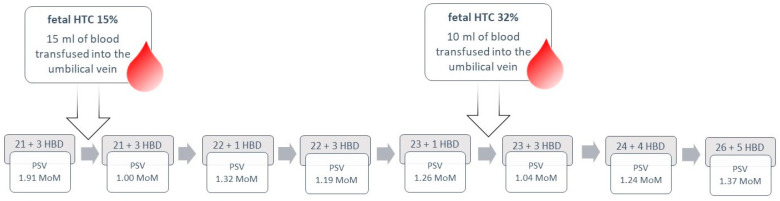
Case 7: Doppler results and treatment strategy presented in a flowchart.

The patient was discharged from the hospital in good general condition, with a PSV MCA of 39.00 cm/s (1.24 MoM) recorded on the day of discharge. Fetal hydrops showed signs of improvement, with only a minimal amount of fluid observed in the fetal abdominal cavity. Doppler results and the timing of intrauterine transfusions are illustrated in Figure 8.

Three weeks after the last intrauterine transfusion, the patient underwent a follow-up ultrasound examination, revealing almost complete resolution of fetal hydrops, with only a small amount of fluid remaining in the pericardium. Fetal echocardiography detected mitral valve regurgitation (Figure 7b). The PSV of the MCA measured 47.57 cm/s (1.37 MoM), which was within normal limits. The estimated fetal weight was 920 g, appropriate for the gestational age.

No further intrauterine transfusion was deemed necessary.

### 3.8. Case 8

A 29-year-old patient was urgently admitted to the hospital during the 26th week of her first pregnancy due to fetal hydrops of unknown origin. The first-trimester and anatomy scans conducted at the 21st week showed no abnormalities or elevated risks for aneuploidies or preeclampsia. The patient reported that her husband, who works with children, had a cold-like infection 14 weeks prior, and she experienced similar symptoms 2 days later.

Upon admission, ultrasound confirmed fetal hydrops characterized by pericardial effusion, ascites, and skin edema. Additionally, impaired heart contractility with significant mitral and tricuspid regurgitation was observed. Evaluation of the ductus venosus blood flow revealed an increased pulsatility index (>99th percentile). Doppler assessment of the middle cerebral artery indicated fetal anemia, with a peak systolic velocity (PSV MCA) of 86.0 cm/s, corresponding to 2.46 multiples of the median for the 26th week of gestation. No other fetal abnormalities were detected during the ultrasound examination.

The differential diagnosis for severe fetal anemia included hemolytic anemia due to alloimmunization, fetomaternal hemorrhage, and parvovirus infection. Laboratory results ruled out the presence of antibodies against red blood cells in maternal serum, and no fetal red blood cells were detected in the maternal circulation. However, antibodies against parvovirus B19 were present.

On the day of admission, CTG indicated severe fetal distress, necessitating immediate delivery due to the imminent risk of intrauterine fetal demise. A male neonate was delivered in critical condition, weighing 1380 g, with an Apgar score of 0, 0, 0, 1. Due to fetal hydrops, there were challenges with intubation and chest compressions. A blood sample taken 30 min after delivery from the umbilical vein showed a pH of 6.7 and an undetectable hematocrit. Sadly, the neonate passed away within the first hour due to cardiorespiratory failure.

## 4. Discussion

Parvovirus B19 infection during pregnancy can cause serious complications, including intrauterine fetal death. Although most infections in pregnant women are asymptomatic, a confirmed parvovirus B19 infection should not be underestimated. It necessitates close monitoring of the fetus for signs of transplacental virus transmission. Early diagnosis and timely treatment are crucial for effectively managing parvovirus B19 infection in the fetus, especially when parvovirus B19 epidemy is recognized, as we observed this year in our region.

Outbreaks of infections occur periodically, typically in the spring. Every few years, there is a notable surge in the number of infections. During epidemic years, the incidence of infections during pregnancy rises, leading to a higher rate of complications such as fetal hydrops or intrauterine fetal death [14]. The cases presented in this study appear to have an epidemic character, as this was the highest number of parvovirus B19-related pregnancy complications reported in this hospital within such a short period over the past decade.

Given the absence of a vaccine for parvovirus B19, it is crucial to assess the risk of infection in pregnant women and identify those at high risk, particularly during epidemic seasons. Pregnant women who are at high risk of exposure to parvovirus B19, such as nursery school teachers or multiparous women with preschool-age children, should have their serological status evaluated [15]. Although routine testing for parvovirus B19 during pregnancy is not recommended, we suggest considering serological testing in patients at high risk for parvovirus B19 infection, particularly during epidemic seasons. In this study, most patients had risk factors for parvovirus B19, and delays in testing led to delayed intrauterine transfusion or fetal death before treatment, resulting in poor obstetric outcomes.

IgM antibodies are detectable about a week after exposure and can persist in the serum for up to 140 days [16]. IgG antibodies appear several days after IgM and persist for years, providing lasting immunity [17]. If a past infection is confirmed by the presence of IgG antibodies and the absence of IgM antibodies, contact with parvovirus B19 will not negatively affect the pregnancy, as only primary infection impacts the fetus. The absence of both parvovirus B19 IgG and IgM antibodies suggests that the patient is susceptible to infection, warranting increased supervision during pregnancy. In cases of fetal hydrops, determining the mother’s serological status may not suffice to confirm the infection, as IgM antibodies might no longer be detectable. In such cases, amniocentesis to detect B19 DNA in amniotic fluid or fetal blood sampling during cordocentesis may be necessary for diagnosis [18].

The presence of IgM antibodies and the absence of IgG antibodies indicate an acute infection, with the virus capable of being transmitted to the fetus via the placenta. The risk of transplacental transmission is estimated to be up to 33% [19].

Most fetal infections are asymptomatic and resolve spontaneously. However, signs of infection in the fetus, particularly fetal hydrops, are strongly associated with adverse neonatal outcomes [18,20]. Our study confirms that fetal hydrops due to confirmed parvovirus B19 infection is highly associated with intrauterine fetal death, as all untreated cases resulted in fetal demise.

Based on our observations, we recommend initiating PSV MCA monitoring 2 weeks after confirmed contact with an individual infected with parvovirus B19. If the exposure date is unknown, monitoring should begin immediately upon receiving a positive serological result for parvovirus B19 infection. If an acute infection is confirmed, ultrasound examinations with measurements of the middle cerebral artery peak systolic velocity (MCA PSV) should be performed every 1 to 2 weeks for up to 12 weeks after possible exposure to the virus [11]. If there is an upward trend in MCA PSV measurements or any signs of fetal hydrops, the patient should be referred to a tertiary care center for consultation with a maternal-fetal medicine specialist [21].

Measuring the peak systolic velocity (PSV) in the fetal middle cerebral artery (MCA) is a non-invasive diagnostic tool for detecting fetal anemia. A value over 1.5 multiples of the median (MoM) supports the diagnosis of severe fetal anemia. This ultrasonographic parameter correlates with hemoglobin concentration and hematocrit levels, making it useful for assessing the severity of anemia and the effectiveness of treatment [22]. Testing fetal blood samples collected during cordocentesis is the most accurate method to determine the severity of fetal anemia. However, adding the measurement of PSV in the MCA to the diagnostic process allows for the avoidance of invasive procedures, which carry some risk of complications. Recent data estimate the fetal loss rate associated with cordocentesis at 1%, although this rate depends on the experience and training level of the person performing the procedure [23,24,25]. The diagnostic capabilities of MCA PSV measurement are limited, and its accuracy decreases with the number of performed intrauterine transfusions [26]. However, this study demonstrates that measuring PSV in the MCA remains useful even after multiple intrauterine transfusions. Decisions regarding further invasive procedures should be based on fetal Doppler and ultrasound results, including a decrease in the severity of hydrops.

The occurrence of fetal hydrops is associated with a high rate of intrauterine death. Despite isolated cases of spontaneous resolution of hydrops [27], there is strong evidence that intrauterine transfusion improves prognosis and increases fetal survival [18,28,29]. In our study, no cases of spontaneous resolution of fetal hydrops were observed, and all fetuses with hydrops that did not receive intrauterine transfusion resulted in fetal demise. Therefore, our study confirms that intrauterine transfusion is essential for improving fetal survival in cases of parvovirus B19 infection. Furthermore, our research indicates that a decrease in the severity of hydrops in fetuses infected with parvovirus B19 is associated with a more favorable prognosis. This is consistent with the findings of Odibo et al. [30], which demonstrated that resolution of fetal hydrops following intrauterine transfusion is associated with advantageous pregnancy outcome. The decrease in the severity of fetal hydrops supports the effectiveness of the treatment and should be taken into account when deciding whether to perform subsequent intrauterine transfusions. In cases of severe fetal hydrops, cordocentesis should be considered to determine the fetal blood count before the serological status of the pregnant woman is determined.

Proper monitoring of pregnancies complicated by parvovirus infection, early detection of ultrasound abnormalities, and timely initiation of treatment are crucial for increasing fetal survival. Moreover, each case of fetal hydrops should be screened for parvovirus B19 infection. In three of the eight cases presented in this study, the diagnosis was made too late, resulting in a delay in intrauterine transfusion and subsequent fetal demise. Consultation should not be delayed because prompt initiation of treatment, such as intrauterine transfusion, is crucial for fetal survival.

The risk of complications associated with intrauterine transfusion (IUT) before the 20th week of pregnancy is higher than that of IUT performed later in pregnancy, according to the literature. However, the survival rate of fetuses transfused in the early second trimester can reach up to 80%, supporting the feasibility of IUT in the early weeks of pregnancy [31]. Kempe et al. [32] described two cases of successful intrauterine transfusion performed in the first trimester. In our study, the earliest intrauterine transfusion was performed at the 20th week of pregnancy, yielding favorable outcomes. Earlier transfusions might improve obstetric outcomes and reduce the incidence of intrauterine fetal death.

Although intrauterine transfusion improves prognosis and increases fetal survival rates, fetuses with advanced hydrops often respond poorly to treatment. In these cases, myocarditis caused by parvovirus B19 may be a critical factor affecting fetal survival [10]. Heart failure secondary to myocarditis may explain why fetal hydrops does not improve or even worsens. Blood administered during a transfusion may exacerbate the fetal circulatory system’s function, leading to fetal demise. Based on one of the cases in our study, leukocytosis detected in the cordocentesis blood count appears to be a strong indicator of myocarditis and is associated with a poor prognosis.

Parvovirus B19 infection affects not only the fetus but also the mother. One of the rare complications of parvovirus B19 infection, with serious consequences for maternal health and life, is mirror syndrome. The mechanism responsible for the development of mirror syndrome is unknown; however, it is most likely the result of endothelial damage caused by toxic placental factors released by the thickened, ischemic placenta. An abnormally high sFlt-1/PlGF ratio in patients with mirror syndrome, which is also observed in preeclampsia, and similar symptoms of both diseases may suggest a common pathophysiologic origin for these conditions [33]. If an abnormal placental image is detected in an ultrasound examination and parvovirus B19 infection is confirmed, detailed diagnostics should be conducted for diseases associated with placental ischemia.

It is hoped that the cases described in this study will contribute to a better understanding of the course of parvovirus B19 infection in pregnancy and help determine the best treatment strategies.

## 5. Conclusions

1. The risk of fetal death is highest if the infection occurs before the 20th week of pregnancy and is significantly greater in hydropic fetuses. Early diagnosis and timely decision-making regarding intrauterine transfusion are crucial for increasing fetal survival, especially in the early second trimester when the risk of intrauterine fetal death is highest.

2. Intrauterine transfusion is the gold standard in the treatment of parvovirus B19-induced fetal anemia and can be safely performed even before 20 weeks using an intravascular approach. The choice of blood transfusion method should be made based on the conditions during the procedure and the experience of the person performing the transfusion. In cases of severe fetal hydrops, the risk of fetal death appears to outweigh the risks associated with performing intrauterine transfusion.

3. Our observations indicate that Doppler analysis (PSV MCA) is an effective tool for monitoring the degree of fetal anemia after intrauterine transfusion (IUT), though improvement in this parameter following intrauterine transfusion does not necessarily ensure the procedure’s success and favorable obstetric outcomes. Therefore, performing the intrauterine blood transfusion to treat infected fetuses is always a worthwhile intervention.

## Figures and Tables

**Table 1:** A summary of the principal characteristics of the cases presented, encompassing gestational age (GA) at admission, ultrasound findings, peak systolic velocity of the middle cerebral artery (MCA PSV) upon admission, serological test outcomes, initial fetal hematocrit levels, the occurrence of intrauterine transfusion, and the pregnancy outcome.

	GA at Admission	Ultrasound Abnormalities	PSV MCA at Admission	Serological Test Results	Initial Fetal Hematocrit	Intrauterine Transfusion	Pregnancy Outcome
Case 1	15	skin edema (++) ascites (++) pleural effusion (+) pericardial effusion (+)	-	IgG (+) IgM (+)	-	no	intrauterine fetal death
Case 2	23	skin edema (+++) ascites (+++) pleural effusion (+++)	65.31 cm/s 2.17 MoM	IgG (+) IgM (+)	-	no	intrauterine fetal death
Case 3	25	skin edema (+++) ascites (+++) pleural effusion (+++) pericardial effusion (+)	63.00 cm/s 1.94 MoM	IgG (+) IgM (+)	-	no	intrauterine fetal death
Case 4	20	skin edema (+++) ascites (+++) pleural effusion (+++) cardiomegaly	59.4 cm/s 2.35 MoM	IgG (+) IgM (+)	unknown	yes	intrauterine fetal death
Case 5	23	thickening of placenta skin edema (+) ascites (+) pericardial effusion (+) cardiomegaly	78.00 cm/s 2.6 MoM	IgG (+) IgM (+)	10.9%	yes	neonate’s death 5 days after delivery (delivery at 26th week of pregnancy)
Case 6	24	skin edema (+++) ascites (+++) pleural effusion (+++) pericardial effusion (+)	89.00 cm/s 3.02 MoM	IgG (+) IgM (+)	6.1%	yes	total resolution of hydrops live pregnancy
Case 7	21	skin edema (+) ascites (++) pericardial effusion (++) mitral valve regurgitation	52.00 cm/s 1.91 MoM	IgG (+) IgM (+)	15.3%	yes	total resolution of hydrops live pregnancy
Case 8	26	skin edema (+++) ascites (+++) pericardial effusion (++)	86.00 cm/s 2.46 MoM	IgG (+) IgM (−)	unknown	no	neonate’s death 1 h after delivery (delivery at 26th week of pregnancy)

## Data Availability

The original contributions presented in the study are included in the article, further inquiries can be directed to the corresponding authors.
